# Effects of Curcumin on Aging: Molecular Mechanisms and Experimental Evidence

**DOI:** 10.1155/2021/8972074

**Published:** 2021-10-13

**Authors:** Afsane Bahrami, Fabrizio Montecucco, Federico Carbone, Amirhossein Sahebkar

**Affiliations:** ^1^Clinical Research Development Unit of Akbar Hospital, Mashhad University of Medical Sciences, Mashhad, Iran; ^2^Clinical Research Unit, Imam Reza Hospital, Faculty of Medicine, Mashhad University of Medical Sciences, Mashhad, Iran; ^3^IRCCS Ospedale Policlinico San Martino Genoa-Italian Cardiovascular Network, 10 Largo Benzi, 16132 Genoa, Italy; ^4^First Clinic of Internal Medicine, Department of Internal Medicine, University of Genoa, 6 viale Benedetto XV, 16132 Genoa, Italy; ^5^Biotechnology Research Center, Pharmaceutical Technology Institute, Mashhad University of Medical Sciences, Mashhad, Iran; ^6^Applied Biomedical Research Center, Mashhad University of Medical Sciences, Mashhad, Iran; ^7^School of Pharmacy, Mashhad University of Medical Sciences, Mashhad, Iran

## Abstract

Aging is characterized by a progressive inability to maintain homeostasis, self-repair, renewal, performance, and fitness of different tissues throughout the lifespan. Senescence is occurring following enormous intracellular or extracellular stress stimuli. Cellular senescence serves as an antiproliferative process that causes permanent cell cycle arrest and restricts the lifespan. Senescent cells are characterized by terminal cell cycle arrest, enlarged lysosome, and DNA double-strand breaks as well as lipofuscin granularity, senescence-associated heterochromatin foci, and activation of DNA damage response. Curcumin, a hydrophobic polyphenol, is a bioactive chemical constituent of the rhizomes of *Curcuma longa* Linn (turmeric), which has been extensively used for the alleviation of various human disorders. In addition to its pleiotropic effects, curcumin has been suggested to have antiaging features. In this review, we summarized the therapeutic potential of curcumin in the prevention and delaying of the aging process.

## 1. Introduction

Aging is identified by a progressive inability to maintain homeostasis, self-repair, renewal, performance, and fitness of different tissues with advancing age [1]. The picture of aging is characterized by genetic and environmental factors ultimately leading to gradual but persistent reduction in cellular proliferation, abnormal oxygen metabolism, and structural instability [2]. A complex gene network contributes to organism lifespan by regulating several critical pathways including protein synthesis and catabolism, energy metabolism, redox balance, intracellular communication, DNA repair, inflammation, cellular senescence, and death [3]. The aging process also involves the vascular system. In this context, cell senescence involving either endothelial cells (ECs) or vascular smooth muscle cells (VSMCs) [4] determines structural and functional alterations resulting in development of endothelial dysfunction [5]. Previous researches identified several molecules and signaling pathways involved in the aging process: among them, growth hormone (GH)/insulin-like growth factor 1(IGF1)/forkhead box O (FOXO) pathway, target of rapamycin (TOR)/ribosomal S6 kinase (S6K), sirtuins (Sirts), p38 mitogen-activated protein kinase (MAPK), and AMP-activated protein kinase (AMPK) [6–8]. Despite many efforts in clarifying the biology of aging and its cellular and molecular mechanisms, standardized biomarkers and therapeutic targets are scarce. Only several senotherapeutics, agents which inhibit senescence (senomorphics) and selectively kill senescent cells (senolytics), have been proposed. Senolytics are drugs that particularly target senescent cells through promoting the apoptosis of senescence [9–11].

In this field of research, there is a growing interest towards the natural compound curcumin (CUR; diferuloylmethane), which is known as an active therapeutic compound against various human disorders owing to its numerous pharmacological actions [12–17]. In light of this, research groups worldwide are attempting to clarify biological pathways, pharmaceutical properties, and potential clinical application of CUR [18]. In this narrative review, we will summarize the therapeutic potential of CUR, especially focusing on prevention and delaying of the aging process.

## 2. Hallmarks of Aging

### 2.1. Oxidative Stress

A prooxidant environment certainly contributes to the aging process by sustaining oxidative modifications of cellular molecules [19–21]. Targets of oxidative stress (OS) include structural damage in cellular macromolecules such as nuclear and mitochondrial DNA, proteins, and lipids [22]. Nevertheless, the “free radical theory of aging” is no longer considered a primitive causal pathway. Free radicals and related oxidants are a subset of stressors with which all living beings must cope with over their lifespans. Rather, the concept of “defective adaptive homeostasis” better describes how aging organisms fail to dynamically expand the homeostatic range of stress defense and repair systems. Indeed, many signal transduction pathways contribute to best fit cellular response to a particular need.

### 2.2. Cellular Senescence

Cellular response to stressors includes three distinctive cellular processes: apoptosis, autophagy, and senescence [23–25]. The latter (from the latin term “senex”: growing old) occurs in response to enormous intracellular or extracellular stress stimuli [26]. Cellular senescence was firstly described by Hayflick and Moorhead [27] as an antiproliferative process leading to permanent cell cycle arrest lifespan reduction [25]. Such effect on the biological clock (Hayflick limit) is generally associated with progressive telomere attrition/dysfunction [28, 29], loss of proteostasis, induction of genes located in the INK4a/ARF locus [30], aberrant oncogene activation, DNA damage during cell division/replication, and apoptosis-resistance [31]. Leading mediators of cellular senescence include the p16^INK4a/Rb^ and tumor suppressor p53/p21 ^CIP1/WAF1^ families of cyclin-dependent kinase (CDK) [32]. Senescent cells endure futile growth, hypertrophy, and hyperfunctions, together with generation and release of inflammatory mediators named senescence-associated secretory phenotype (SASP) [33, 34]. SASP includes multiple inflammatory elements such as interleukin- (IL-) 6, IL-8, IL-1, tumor necrosis factor-*α* (TNF-*α*), nuclear factor kappa B (NF-*κ*B), and growth factors like insulin-like growth factor- (IGF-) 1, platelet-derived growth factor (PDGF), vascular endothelial growth factor (VEGF), and basic fibroblast growth factor (bFGF) [35, 36].

Alongside SASP, the core event in cellular senescence cell nucleus are the disturbances in DNA repair mechanisms, which determine DNA double-strand breaks senescence-associated heterochromatin foci (SAHF), terminal cell cycle arrest with resistance to apoptosis, and loss of regeneration/resilience [37]. Additional features include enlarged lysosomes, overexpression of senescence associated *β*-galactosidase (SA-*β*-gal), and lipofuscin granularity as well. A relevant feature of aging is chronic low grade inflammation, referred to as “inflammaging” which is the age-related inflammatory status, results from immunosenescence, as it is found to be associated with the majority of age-related diseases sharing an inflammatory basis [38]. Together with immunological elements, cellular senescence and the SASP are the major contributors to inflammaging.

That cellular senescence may have a causative role in organismal aging [39]. During aging, senescent cells are possibly persistent, activated by random molecular damage and related with the activation of a DNA damage response [40]. The collection of senescent cells in animal organs may be involved in the aging process through reducing the renewal competence of tissues [30] and/or via reforming the tissue structure and activity by secretion of matrix metalloproteinases, epithelial growth factors, and inflammatory mediators which could intrude with the tissue microenvironment [41]. Therefore, tissue homeostasis will be compromised which finally will result to aging.

### 2.3. Sirtuins

Sirtuins are NAD+-dependent deacetylases, ubiquitously distributed in either prokaryote or eukaryote cells [42]. In mammalians, 7 *Sirt* genes (Sirt1 to Sirt7) have been identified. Sirt1 belongs to the class III histone deacetylases (HDAC) with activity on various transcriptional factors (TFs), histones, and cytoplasmic proteins with acyl-lysine residues [43]. Antiaging properties of Sirt1 include the suppression of a typical senescent secretome through epigenetic gene modulation [44]. However, the antiaging effects of Sirt1 are far from being elucidated, potentially ranging from mitochondrial respiration to stress modulation, energy expenditure, and p53 deacetylation [37, 45].

## 3. Curcumin

Due to their ubiquitous distribution in food, phytochemicals attract more attention because of their obvious safety. Accumulating evidences reported how phytochemicals that can extend lifespan also enhance wellness in different heterotrophic organisms [46–49]. The hydrophobic yellow polyphenol CUR is a bioactive chemical constituent of the rhizome of *Curcuma longa Linn*, extensively used in cooking as food coloring and preservative. CUR is the main chief ingredient of turmeric representing nearly 2–5% of the plant [50]. Toxicity studies claimed it is a safe compound agent even at high doses [51]. Concerning effectiveness, several lines of evidence highlighted a pleiotropic potential of CUR towards several human diseases, such as malignancies, skin and immune-related disorders, cardiovascular diseases, pulmonary and renal fibrosis, nonalcoholic fatty liver disease (NAFLD), fatigue, neuropathic pain, bone and muscle loss, neurodegenerative disease, ocular diseases, leprosy, osteoporosis, leishmaniosis, and HIV infection [52–57]. Pleiotropic functions of CUR mainly rely on the inhibition of I*κ*B kinase (IKK) phosphorylation [58] and the consequent suppression of the nuclear translocation of the NF-*κ*B p65 subunit [59]. As an alternative epigenetic modulator, CUR also enhances Sirt1 expression at both mRNA and protein levels, ultimately resulting in the suppression of histone acetyltransferase (HAT) activity and increased NAD^+^/NADH ratio [60, 61]. With the same mechanism, CUR modulated the expression of several types of microRNAs [62–65]. Through those mechanisms, CUR supplementation in human melanoma cells induces growth arrest in the G2/M phase and then apoptosis [66]. Other studies also reported that CUR may target oncogene expression, angiogenesis, invasion, and metastatic dissemination [67, 68] by interfering with several other intracellular pathways including hypoxia-inducible factor-1 *α* (HIF-1 *α*), mammalian sterile 20-like kinase 1 (MST1), enhancer of zeste homolog 2 (EZH2), platelet-derived growth factor (PDGF) receptor binding, Wnt/*β*-catenin, transforming growth factor beta (TGF-*β*), Sonic Hedgehog, Notch, and phosphatidylinositol 3-kinase (PI3K)/Akt/mammalian target of rapamycin (mTOR) cascade [69–71]. Alongside with antitumorigenic activity, CUR was also shown to induce antimicrobial, antioxidant, antiglycemic, antiseptic, and analgesic effects [72–74]. This “pleiotropic” potential may be ascribed to the potent metal-chelating effects of CUR, which include the scavenging of the superoxide anion, hydroxyl radical, singlet oxygen, and nitrogen dioxide [75, 76]. In line with this, other studies demonstrated that CUR may reduce levels of malondialdehyde (MDA), protein carbonyls, thiols, and nitrotyrosines. With regard to inflammation, CUR stimulates a xenobiotic response with upregulation of defense genes (e.g., phase II enzymes and hemeoxygenase-1 [HO-1]) [77] and suppression of proinflammatory transcription factors (e.g., activator protein-1 [AP1]) and cytokines (e.g., TNF-*α*, IL-1b, IL-6, IL-8, and monocyte chemotactic protein 1 [MCP-1]), signal transducer activator of transcription (STAT), peroxisome proliferator-activated receptor-*γ* (PPAR-*γ*), activating transcription factor 3 (ATF3), C/EBP homologous protein (CHOP), and the inducible inflammatory enzymes cyclooxygenase- (COX-) 2 and metalloproteinases [78].

Finally, as observed in human skin fibroblasts, CUR may activate cellular stress response by interacting with the thiol-disulfide redox system. Such stress determines a rise in cellular GSH amounts via HO-1 and nuclear factor E2-related factor 2 (NRF2) signaling [79], ultimately improving cellular antioxidant defenses [80, 81]. Moreover, several studies indicated that CUR and may be used as senolytic and anti-inflammatory agents for senescent cells [82, 83]. For instance, a CUR analog, EF24, promoted senescent cell apoptosis and showed protection effect against ionizing-stimulated senescent cells [83].

## 4. Effect of Curcumin on Aging/Longevity

### 4.1. Vascular Aging

**Figure 1:**
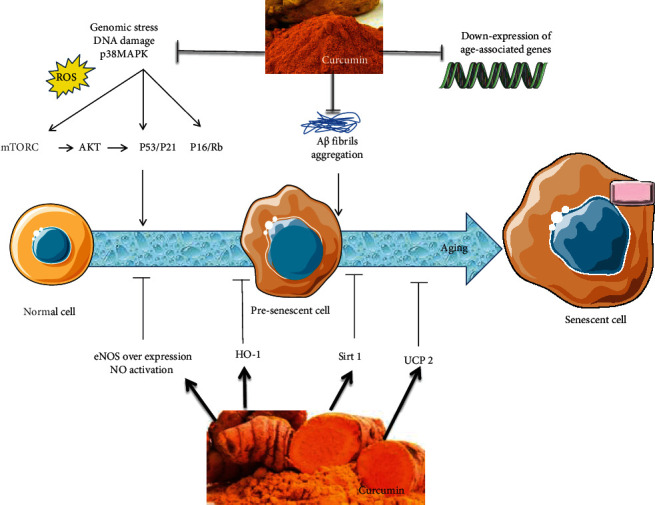
Mechanisms by which curcumin modulate aging process and senescence. Curcumin inhibited OS-stimulated p38MAPK activation, A*β* fibril aggregation, and expression of age-associated genes (*dInR*, *ATTD*, *Def*, *CecB*, *DptB*, *mth*, *thor*, *InR*, and *JNK*), although curcumin induced eNOS, NO, Sirt1, HO-1, and UCP2 expression. Curcumin also mitigates the SASP and its aging-induction consequences of senescent cell. Abbreviations: A*β*: amyloid-*β*; eNOS: endothelial nitric-oxide synthase; HO-1: hemeoxygenase-1; mTORC 1: mammalian/mechanistic target of rapamycin complex 1; NO: nitric oxide; ROS: reactive oxygen species; SASP: senescence-associated secretory phenotype; Sirt: sirtuins; UCP2: uncoupling protein 2.

Further enhancing a wide spectrum of activity, growing evidence indicates CUR as a promising antiaging agent (Table 1; Figure 1) [84, 85]. The effects of CUR feeding have been largely investigated in animal models, unanimously reporting a suppression of intermediated oxidative stress (e.g., lipoxygenases [LPO], MDA, lipofuscin granules, and NO) and inflammation [3, 86]. By chelating nitrogen dioxide (NO_2_), CUR administration in mice significantly attenuates nitric oxide- (NO-) associated vascular endothelial dysfunction and generation of advanced glycation end-products (AGEs), leading determinants of age-related large elastic artery stiffening [87]. As an additional mechanism, CUR fixes lysosomal membranes and reduces the function of lysosomal acid hydrolases, thus preventing the aberrant deposition of different connective tissue components in aging endothelium. A similar upgrade in endothelial function was also observed in postmenopausal women after eight weeks of treatment [88], whereas in elderly with diabetes and cardiomyopathy, CUR mitigated hypertrophy in the aging heart via suppression of p300, the global transcription activator [89]. Beneficial effects of CUR on vascular aging also concern the development of age-related macular degeneration (AMD), one of the most important causes of blindness in elderly [90, 91]. CUR remarkably increases the viability of retinal pigment epithelial cells (RPECs) modulating their proliferation apoptosis and OS [92]. Overall, those evidences suggest potential application of CUR as an innovative approach to AMD, as for other ocular diseases (e.g., ocular dryness, conjunctivitis, uveitis, pterygium, and glaucoma) [93]. Even CUR has been found to prevent the development of cataract in diabetic rats by decreasing AGE accumulation and serum LPO [94, 95]. Aging-associated cerebrovascular endothelial dysfunction with consequent chronic cerebral ischemia also plays a critical role in stroke, as well as in cerebral amyloid angiopathy, cognitive impairments, and neurodegenerative disorders [96–98]. One of the main pathological mechanisms behind this effect is the generation of ROS, due to the suppression of mitochondrial uncoupling protein 2 (UCP2) [99] and the downregulation of AMPK. CUR reverses those effects in cultured ECs, whereas in experimental models, prolonged CUR feeding decreased ROS generation and promoted cerebrovascular endothelium-dependent relaxation, finally leading to improved cerebrovascular function [100–103]. Neuroprotective effects of CUR due to UCP2 overexpression suppression especially target hippocampal neurogenesis in the CA1 area, thus affecting spatial learning and memory. CUR also prevents detrimental effects of chronic cerebral hypoperfusion by maintaining cholesterol homeostasis. CUR also contributes to maintain cholesterol homeostasis, otherwise upset by chronic cerebral ischemia. Indeed, CUR promotes cholesterol efflux through the ATP-binding cassette transporter A1 (ABCA1) and the pathway involving apoA-I and the liver X receptor (LXR)/retinoic X receptor (RXR) [104].

### 4.2. Cognitive Impairments

With similar mechanisms, the reduction of circulating antioxidants is tightly associated with memory loss and cognitive impairment in the elderly [105]. It is then not surprising that CUR has been reported to improve neuropsychological functions. CUR has several inhibitory effects on combining aging and Alzheimer's disease pathophysiology, such as the suppression of amyloid precursor protein (APP) and A*β* synthesis and the overexpression of *ApoE* and *Nrf2* gene, as well as the prohibition of p-mTOR and p-NF-*κ*B [106, 107]. CUR prevents D-gal-induced brain aging and cognitive impairment through increments of antioxidant enzymes and inhibition of apoptosis [108]. Beneficial effects of CUR on mental abilities and functional capacities are associated with a LPO reduction in brain tissue [109], especially in the hippocampal area. CUR improves the redox state in this area and prevents the decline of hippocampal long-term potentiation by maintaining synapse input specificity [110, 111]. Recently, Olesen et al. described that the dysfunction of synaptic mitochondria of the hippocampus causing memory loss during aging. They showed that curcumin feeding significantly improved integration and activity of the synaptic mitochondrial of the hippocampus, inhibiting mitochondrial swelling and enhancing the production of synapses surrounding the mitochondria in mice [112].

### 4.3. Evidence from Experimental Models

#### 4.3.1. Study of Longevity in Drosophila melanogaster and Caenorhabditis elegans

Drosophila melanogaster (*D. melanogaster*) and *Caenorhabditis elegans* (*C. elegans*) are widely recognized models for the study of aging processes [113]. In particular, *D. melanogaster* represented a paradigm of experimental gerontology during the last century [114–118] because of its complex biology and the ease of rearing and housing as well [119, 120]. More recently, in 1983, Klass isolated the first long-lived mutants of *C. elegans* [121], which rose to become a promising model for aging investigations due to the small size, anatomical simplicity, small genome, short life cycle, and inexpensive laboratory manipulation [122]. In *C. elegans*, longevity is widely determined by the expression of the Age-1 gene [123, 124]. As one of the main elements in the insulin/insulin-like growth factor-1 signaling (IIS) axis, Age-1 is a subunit of phosphoinositide 3-kinase (PI3K), which suppresses DAF-16 action [123–125]. Suppression of the IIS pathway activates the downstream gene DAF-16, which in turn promotes the transcription of genes associated with longevity, metabolism, and response to cellular stress [126–128]. In line, increased lifespan may also be obtained through TOR inhibition, another DAF-16 suppressor [129, 130]. By sharing the same downstream signaling of DAF-16, also the *FOXO3 A* gene is involved in lifespan extension, cell growth, and stress response through a direct activity on DNA repair and transcription involving p21/p53 and *β*-catenin pathways [131–133]. Noteworthy, FOXO has a multistep regulation involving not only IGF-1 but also NAD+/Sirt1, 5′AMPK, and OS, all known as aging genes [134]. Due to these similarity with human beings, *C. elegans* became a genetic model organism already in 1965. Multiple pharmacological interventions have been found to prolong the survival of *D. melanogaster* and *C. elegans* [135–137]. Also, CUR was shown to increase the fecundity, reproductive lifespan, and child viability of *D. melanogaster* [85]. It has been shown that CUR supplementation at the larval stage of *D. melanogaster* elevated the developmental duration and longevity of adult Drosophila possibly through epigenetic programming of the pace of life [138].

CUR-mediated increased longevity was observed in two distinctive strains of *D. melanogaster* (Canton-S and Ivies flies) as a result of the delayed expression of aging genes (e.g., methuselah (*mth*), *thor*, insulin receptor [*InR*], and c-jun N-terminal kinase [*JNK*]), improved locomotion, and chemoprevention as well [139]. CUR was also shown to reduce OS, DNA damage, and number of mutagenic phenotypes induced via high-dose ionizing irradiation. These effects may be ascribed to ROS scavenging and transcriptional regulation of OS-related genes, which mainly involves *γ*H2Ax, a histone protein belonging to the H2A family and involved in DNA damage response [140–142]. Also, *in vivo* experiments on CUR-fed diets (0.5 and 1.0 mg/g of diet) were effective in extending the average lifespan in both females (6.2% and 25.8%, respectively) and males (15.5% and 12.6%, respectively), and this effect could be more likely attributed to the overexpression of Mn-SOD and CuZn-SOD genes and the downregulation of aging genes associated with the TOR pathway including *Drosophila* insulin receptor (*dInR*), attacin-D (*ATTD*), defensin (*Def*), cecropin B (*CecB*), and diptericin B (*DptB*) genes [143, 144]. Also, in *C. elegans*, CUR effectively improves lifespan and aging by lowering intracellular ROS and lipofuscin. The effects of CUR on *C. elegans* longevity are manifested by body size and pharyngeal pumping rate but not reproduction ability. Further studies revealed that the long-lived phenotype induced by CUR was maintained in mev-1 and daf-16 mutants but lost in osr-1, sek-1, skn-1, unc-43, mek-1, sir-2.1, and age-1 ones [145]. This evidence indicates that CUR would exert its effects independently of the Age-1-DAF-16 pathway but rather through other constituents of the IIS pathway. With regard to cognitive impairment, the *in vivo* experiment demonstrated that CUR can improve learning and memory also reducing A*β* plaque formation in the context of Alzheimer disease (AD) [146]. *D. melanogaster* is a promising animal model for research in AD [147]. By increasing amyloid fibril conversion, CUR reduces the generation of prefibrillar/oligomeric species of A*β*, ultimately protecting against neurotoxicity [148]. The human *β*-amyloid precursor cleavage enzyme (BACE-1) is another critical enzyme targeted by CUR [149, 150] in the *D. melanogaster* model of AD [150].

#### 4.3.2. Studies of Cell Senescence: Evidence from Mice and Rats

High doses of CUR (2.5-10 *μ*M) were shown to trigger senescence in cancer and vascular cells [151]. On the other hand, low doses of CUR (0.1 and 1 *μ*M) failed to prevent early senescence in doxorubicin-treated (VSMC) and even slightly accelerated replicative senescence in endothelial cells [152]. It is therefore evident how the antiaging effect of CUR does not rely on delayed cellular senescence. As reported by Banji et al., CUR (40 mg/kg) and piperine (12 mg/kg), especially when combined, counteract D-gal-induced senescence in male Wistar rats by targeting OS and lipofuscin deposition, finally leading to higher hippocampal volume and function with improved spatial memory and serotoninergic signaling [153]. Another study even reported how long-time CUR therapy may progressively reverse cognitive dysfunction in D-gal-induced senescent mice by delaying the aging process and improving cognitive functions and locomotor activity, as well as restoring the mitochondrial enzyme complex function [154]. In a recent study, CUR supplementation rejuvenates senescence-associated changes in thymus among D-gal-induced senescent mice through promotion of proliferating cells, preventing cells from apoptosis, and enhancing the transcription of the autoimmune regulator (Aire) [155].

CUR feeding (50 mg/kg) was also tested in senescence-accelerated mouse prone (SAMP) mice resulting in increased hippocampal SOD activity as well as upregulation of p-calcium/calmodulin-dependent kinase II (p-CaMKII) in the stratum lucidum and p-N-methyl-D-aspartate receptor subunit 1 (p-NMDAR1) in the hippocampal membrane [156]. Noteworthy, clinical benefits of the CUR analogue PE859 have been recently reported and associated with reduction of A*β* and tau aggregates in the mouse brain [157, 158]. Overall, these findings suggest a role of CUR in improving cognitive difficulties and the expression of hippocampal plasticity-associated proteins. With regard to vascular function, CUR administration significantly mitigated premature senescence in HUVECs, characterized by a reduction of senescence-related *β*-galactosidase-positive cells, cell division, levels of senescence-related protein p21 RNA, OS, and apoptosis. CUR is also associated with enhanced eNOS phosphorylation and NO generation, in addition to upregulating Sirt1 transcription, translation, and enzymatic activity [159]. In light of these mechanisms, diets containing tetrahydrocurcumin (THC), the main metabolite of CUR, were demonstrated to significantly extend mean lifespan in male C57BL/6 mice [160], whereas bisdemethoxycurcumin administration delayed the OS-caused premature senescence via Sirt1/AMPK cascade activation [161]. As recently demonstrated, Sirt1 signaling also mediates the anti-inflammatory effects of CUR in C57BL/6 mice fed with high fat diet [162] in addition to improved myocardial structure and function in streptozocin-induced diabetic mice fed with THC (120 mg/kg/d) [163]. Even more recently, it has been hypothesized that the antiaging effect of CUR may rely on the control of core clock genes on which Sirt1 belongs alongside rBmal1, rCry1, rCry2, rPer1, rPer2, and rRev-erba. CUR treatment in middle aged male Wistar rats restored the phase and daily pulse of rCry1, rCry2, rPer1, and rPer2 as in the young, whereas only rPer1 and partly rBmal1, rCry1, and rCry2 were restored in the old ones [164]. Moreover, it has been shown that CUR mitigated mouse ovarian aging, upgraded embryonic development, promoted oocyte maturation and fertilization via improvement of ovarian hormones, and elevated the amounts of *SIRT1* and *3* genes as well as attenuation of aging-associated oxidative stress and cell death [165]. Besides, CUR can reduce oxidative stress, inflammation status, and lipofuscin deposition in aged rat liver [166].

## 5. Conclusion

Aging and senescence are complex processes leading to organ dysfunction. Despite being permanent, delaying the occurrence of these processes is a reliable target, and CUR might be a promising candidate for this purpose. Nevertheless, evidence from clinical studies on the long-term effects of CUR on age-related pathological events remains largely understudied. While several strategies to enhance the systemic bioavailability of CUR have been suggested, the effects of long-term therapy with such bioavailability-boosted CUR preparations is not fully known, and increased concentrations may even lead to opposite results. Pleiotropic benefits of CUR supplementation involve the control of aging genes, OS, and inflammation in both the vascular system and the central nervous system. Further studies are warranted to clarify the mechanisms of CUR function for potential clinical application.

## Figures and Tables

**Table 1:** Antiaging effect of curcumin.

Compound	Animal model	Effect	Reference
Curcumin	(i) Aged female Wistar rats	(i) Decreasing the MDA and LPO levels in brain tissue	[109]
Curcumin (20, 40, and 80 *μ*M)	(i) Aging RPE cells	(i) Improvement of cell viability (ii) Reducing the apoptosis and OS (iii) Decreasing the expression of apoptosis-related proteins and OS biomarkers	[92]
Curcumin (0.2%)	(i) Male Sprague Dawley rats (ii) UCP2 knockout (UCP2-/-) (iii) Matched wild-type mice	(i) Restoring the impaired cerebrovascular endothelium-dependent vasorelaxation (ii) Promoting eNOS and AMPK phosphorylation (iii) Overexpression of UCP2 and reduction of ROS generation	[103]
Curcumin (0.2%)	(i) Male C57BL/6N mice	(i) Ameliorates age-associated large elastic artery stiffening (ii) Improvement of NO-mediated vascular endothelial dysfunction (iii) Oxidative stress (iv) Decreasing the collagen I and AGEs in the arterial wall	[87]
Curcumin (100 *μ*M)	(i) Wild-type Canton-S flies	(i) Protective effect against radiation damage (ii) Decrement of the amount of protein carbonylation and *γ*H2Ax foci	[142]
Curcumin (100, 200, and 400 mg/kg BW)	(i) Female Wistar albino rats	(i) Increased the NO and MDA levels	[3]
Curcumin (50 mg/kg)	(i) Adult and aging male C57BL/6 mice	(i) Modulation of hippocampal redox status (ii) Restoring aging-related loss of synapse input specificity of HFS-LTP	[110]
Curcumin (50 and 100 mg/kg)	(i) Male Sprague Dawley rats	(i) Improving the spatial learning and memory (ii) Alleviating pathological change (iii) Reduction of the level of MDA (iv) Increment of the activity of SOD (v) Inducing HO-1 protein expression (vi) Increasing the protein levels of UCP2 (vii) Inhibiting OS induced by ischemia	[167]
PE859	(i) SAMP8	(i) Inhibition of A*β* aggregation (ii) Amelioration of cognitive dysfunction (iii) Decrements of the amount of aggregated A*β* and tau	[158]
Curcumin (5 to 100 *μ*M)	(i) HUVECs	(i) Mitigated the H_2_O_2_-induced endothelial premature senescence (ii) Decrements of population of senescence-related *β*-galactosidase-positive cells (iii) Motivating cell division (iv) Dwindling RNA amplification of senescence-related protein p21, OS, and apoptosis (v) Induction of the expression of the phosphorylation of eNOS (vi) Increments of the amount of NO (vii) Stimulation of the transcription, translation, and enzymatic activity of Sirt1	[159]
Piperine (12 mg/kg)+curcumin (40 mg/kg)	(i) Adult male Wistar rats	(i) Improvement of spatial memory and serotoninergic signaling (ii) Decrements of OS and lipofuscin deposition (iii) Higher hippocampal volume (iv) Hippocampal neuroprotection (v) Promotion of cognition (vi) Inhibition of senescence by the free radical quenching	[153]
Curcumin (50 mg/kg)	(i) SAMP8 mice	(i) Narrowing the hippocampal SOD activities (ii) Elevation of the amount of p-CaMKII in the stratum lucidum of hippocampal CA3 and p-NMDAR1 in the hippocampal membrane	[156]
Curcumin (0 to 500 mM)	(i) Two strains of Drosophila (Canton-S and Ives flies)	(i) Protection against oxidative stress (ii) Improvement in locomotion (iii) Modulating the expression of different aging-associated genes, including *mth*, *Thor*, *InR*, and *JNK*	[139]
Curcumin (0 to 200 mM)	(i) Normal-lived Ra strain (*Drosophila*)	(i) Induction of an extended longevity phenotype (ii) Slowing the aging rate (iii) Increases the adult animal's geotactic activity	[144]
Curcumin (0.5 to 1.0 mg/g of diet)	(i) Oregon-R strain (*Drosophila*)	Overexpression of Mn-SOD and CuZn-SOD genes (i) Downexpression of age-associated genes (*dInR*, *ATTD*, *Def*, *CecB*, and *DptB*) (ii) Modulating the gene expression of SOD (iii) Decrements of MDA and LPO	[143]
Galantamine (5 mg/kg) and curcumin (15 and 30 mg/kg)	(i) Old male LACA mice	(i) Postponing aging process (ii) Improving cognitive functions, locomotor activity, and antioxidation (iii) Decrements of acetylcholine esterase activity (iv)Restoring the mitochondrial enzyme complex execution	[154]
Curcumin	(i) Transgenic Drosophila	(i) Increments of amyloid fibril conversion by decreasing the prefibrillar/oligomeric species of A*β*	[148]
Curcumin and disulfiram/gram of media	(i) Male *D. melanogaster*	(ii) Promotion of SOD activity	[168]

## Abbreviations

A*β*: Amyloid-*β*
AGEs: Advanced glycation end-products
*ATTD*: Attacin-D
*CecB*: Cecropin B
*Def*: Defensin
*dInR*: *Drosophila* insulin receptor
*DptB*: Diptericin B
HO-1: Hemeoxygenase-1
HFS: High-frequency stimulation
H2O2: Hydrogen peroxide
HUVECs: Human umbilical vein endothelial cells
GSH: Glutathione
LTP: Long-term potentiation
MDA: Malondialdehyde
NO: Nitric oxide
RPE: Retinal pigment epithelial
p-CaMKII: p-Calcium/calmodulin-dependent kinase II
SAMP8: p-N-Methyl-D-aspartate receptor subunit 1 (p-NMDAR1), senescence-accelerated mouse prone 8
SOD: Superoxide dismutase
UCP2: Uncoupling protein 2.
